# Multi-decadal oscillations of surface temperatures and the impact on temperature increases

**DOI:** 10.1038/s41598-022-24448-3

**Published:** 2022-11-18

**Authors:** Christoph Kalicinsky, Ralf Koppmann

**Affiliations:** Institute for Atmospheric and Environmental Research, University of Wuppertal, Wuppertal, Germany

**Keywords:** Attribution, Atmospheric dynamics

## Abstract

The last IPCC assessment report indicated that natural climate variability could temporarily amplify or obscure anthropogenic climate change on decadal time scales. Here we analyse global mean surface temperatures in terms of such long-period variations. We find two main oscillations, a strong oscillation with a period of about 70 years and an amplitude of about 0.09 K and a quasi-bidecadal oscillation with an amplitude of about 0.06 K. The strong oscillation shows large hemispheric differences. In the Northern hemisphere the period is longer and the amplitude is larger (about 82 years and 0.18 K) compared to the Southern hemisphere (about 47 years and 0.065 K). No obvious hemispheric differences are observed for the quasi-bidecadal oscillation. Such long-period oscillations can strengthen or weaken the temperature increase if the oscillation positively or negatively adds to the underlying long-term trend.

## Introduction

Temperature oscillations in the atmosphere, ocean, and coupled atmosphere–ocean system occur on a variety of time scales, ranging from multi-year to multi-decadal periods. In this work, we focus on long-period oscillations with periods beyond the 11-year solar cycle. One oscillation that is frequently discussed in the literature in this context is a quasi-bidecadal oscillation. Several previous studies report evidence of such an oscillation based on the analysis of meteorological parameters such as temperature, precipitation, and temperature variability. The authors explain the observations in the context of the Hale cycle^1–3^. Furthermore, a quasi-bidecadal oscillation of surface temperatures has also been reported in recent literature^4,5^. In addition to these observations at the surface, a quasi-bidecadal oscillation has also been reported at higher altitudes. It has been observed in the stratosphere^6,7^ and in the mesophere and mesopause region^8–10^. Observations and simulations show that this quasi-bidecadal oscillation has a clear vertical structure with neighbouring regions that behave opposite to each other. These results suggest a periodic vertical shift of the temperature profile as the mechanism for this structure^10,11^.

Oscillations with longer periods in the range of 50–90 years have also been discussed on a global and local scale^4,5,12–16^. Some of these results showing long-period oscillations have been discussed as internal variability of the coupled atmosphere–ocean system. Here, the AMV/AMO (Atlantic Multidecadal Variability/Oscillation) and the IPO/PDO (Interdecadal Pacific Oscillation/Pacific Decadal Oscillation) are the main candidates that can cause variations in surface temperatures and show periods in the two regions of interest (bidecadal and > 50 years)^5,16–23^.

In contrast to the explanation that the observed oscillations are (mainly) natural in origin, other studies suggest that changes in aerosols in the atmosphere cause most of the multi-decadal variability at global and local scales^24,25^.

In our study, we focus on hemispheric and regional differences in the periods and amplitudes of the observed main long-period oscillations, as we believe these aspects are only partially, if at all, discussed in the recent literature. In addition, the question of whether the oscillations are of natural origin or not is still important.

## Long-term trend descriptions

**Figure 1:**
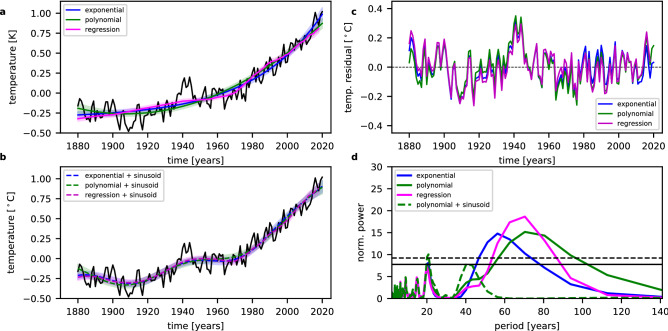
Comparison of different long-term trend descriptions. (**a**) The global annual mean surface temperature anomalies (GISTEMP v4) are shown as black curve. The fits of an exponential function, a second-degree polynomial, and the regression using the radiative forcing are shown in blue, green, and magenta, respectively. (**b**) Same as in (**a**) but with an additional sinusoid in the fits. The coloured areas show the 2σ uncertainties of the fits in all cases. Because of the end of the radiative forcing time series in 2018, the fit using the correlation also ends in 2018. (**c**) The residual temperatures (original temperature time series—fit) for the three different long-term trend descriptions are shown with the same colours as in (**a**) and (**b**). (**d**) Lomb-Scargle Periodogram of the detrended time series of surface temperature anomalies. The different fit functions used for the detrending are shown with different colours. The LSP is evaluated at 278 evenly spaced frequencies in the range f = 1/2 year^−1^ to f = 1/141 year^−1^. The black solid line and the dashed black line display the levels for false alarm probabilities of 5% and 1%, respectively. In the case of the correlation analysis, these levels are slightly lower, because of the shorter length of the time series and the smaller possible maximum peak. However, the difference can hardly be seen.

The global annual mean surface temperatures (GISTEMP v4)^26–28^ are shown in Fig. 1a together with the results for different descriptions of the long-term trend; a regression using the radiative forcing, a second-degree polynomial, and an exponential function (see “Methods” section). The magenta curve depicts the regression using the radiative forcing. Obviously, this curve is very similar to the two other simple descriptions, the exponential function (blue curve in Fig. 1a) and the second-degree polynomial (green curve in Fig. 1a). At the end of the time series, the red curve is very close to the polynomial and at the beginning very close to the exponential function. The largest deviations in terms of the shape occur in the period between about 1940 and 1970. The aerosol loading in the atmosphere largely increased in this time, which led to a change in the aerosol radiative forcing (decrease to more negative values) and thus to a flattening of the increase of the complete radiative forcing^29^. The influence of the long-term trend description on the strongest oscillation is very small. The period derived with the exponential function as description of the long-term trend is slightly smaller than the other two (60.7 years compared to 67.6 (regression) and 70.7 years (polynomial)), but the difference is only slightly larger than the combined 1σ uncertainties, which are in the range of 2.7–3.7 years.

As the radiative forcing time series is only available as global mean value and the results for the regression and the polynomial show a very good agreement at the end of the time series, we mainly concentrate on the second-degree polynomial as a description of the long-term trend.

Figure 1c shows the different residual time series after subtracting the long-term trend for all three cases. All residual time series show a clear long-period oscillation, which is almost identical in all cases. The Lomb-Scargle periodograms (LSP) for all of these residual temperatures show significant peaks in the range of 55–70 years (see Fig. 1d). Figure 1b shows the results for the fits including the long-term trend plus one sinusoid for the strongest oscillation using the same colours as in Fig. 1a. Obviously, all three fits give a reasonable description of the long-term evolution and almost consistent results. When the fit consisting of the polynomial and one sinusoid is subtracted from the original temperature time series and a LSP is calculated again (green dashed line in Fig. 1d), the peak at about 20 years becomes even more pronounced than before. It is now the largest peak in the periodogram with a value above the 1% false alarm probability (FAP) level. Thus, this peak and therefore the oscillation is significant under the assumption that the long-term trend and the long-period oscillation determined before are valid.

## Main oscillations and hemispheric differences

**Figure 2:**
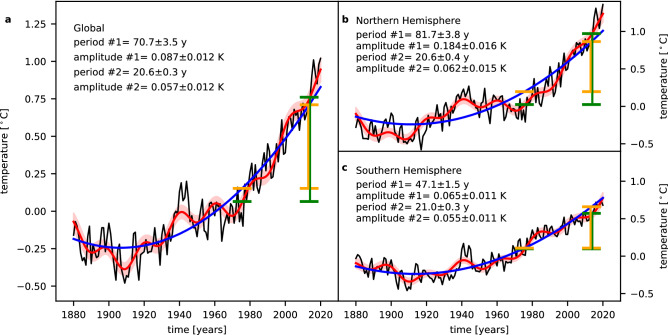
Long-periodic oscillations of global and hemispheric annual mean temperatures (GISTEMP v4). (**a**)–(**c**) The annual mean temperature anomalies are shown as black curves and the fits of a second-degree polynomial plus two sinusoids to these time series are shown as red solid curves. The reddish areas mark the 2σ uncertainty ranges of the fit curves. The blue curves display the second-degree polynomials only. The orange horizontal lines show the mean values in the time intervals 1971–1980 and 2009–2018 for the polynomial and the green horizontal lines show the mean values in the same time intervals for the complete fit curves.

Figure 2a shows the global annual mean surface temperature anomalies and a fit of two sinusoids and a long-term trend to the data. The initial values for the sinusoids are estimated using the LSP in two steps (compare Fig. 1c green solid and dashed curve). The results are shown in Table 1. Also, the results for the exponential function as long-term trend description are listed for comparison. Thus, the two main oscillations occur at periods of about 70 and 20.5 years with amplitudes of about 0.09 and 0.06 K, respectively. We performed the same analysis for the two hemispheric annual mean surface temperature anomalies (Fig. 2b, c and Table 1). Obviously, there are large differences between the two hemispheres with respect to the period and amplitude of the strongest oscillation (#1 in Table 1 and Fig. 2). In the Northern hemisphere (NH) the period is longer and the amplitude is much larger (about 82 years and 0.18 K) compared to the Southern hemisphere (SH) (about 47 years and 0.065 K). Therefore, the oscillation observed for the global mean temperatures is mainly influenced by the NH. The oscillation in the SH leads to a slight reduction of the period and to a larger reduction of the amplitude. However, the global temperature behaviour is much more related to the temperature behaviour of the NH. These clear hemispheric differences are confirmed when we use an exponential function for the long-term trend description.

**Table 1:** Comparison of the results for global and hemispheric annual mean surface temperatures.

	Global		Northern hemisphere		Southern hemisphere	
	Period #1	Amplitude #1	Period #1	Amplitude #1	Period #1	Amplitude #1
Polynomial	70.7 ± 3.5	0.087 ± 0.012	81.7 ± 3.8	0.184 ± 0.016	47.1 ± 1.5	0.065 ± 0.011
Exponential	60.7 ± 2.3	0.081 ± 0.012	73.3 ± 2.3	0.153 ± 0.016	48.8 ± 1.5	0.083 ± 0.013

	Period #2	Amplitude #2	Period #2	Amplitude #2	Period #2	Amplitude #2
Polynomial	20.6 ± 0.3	0.057 ± 0.012	20.6 ± 0.4	0.062 ± 0.015	21.0 ± 0.3	0.055 ± 0.011
Exponential	20.4 ± 0.3	0.057 ± 0.011	20.5 ± 0.4	0.063 ± 0.015	21.0 ± 0.4	0.056 ± 0.013

The periods are given in years and the amplitudes are given in K. All uncertainties are 1σ uncertainties.

In the case of the second largest oscillation (#2 in Table 1 and Fig. 2) there is a remarkable agreement between all results independent of the hemisphere and the long-term trend description. All estimated periods and amplitudes agree within the combined 1σ uncertainties. Thus, there is no hemispheric difference for the second largest oscillation.

## Global distribution of strongest oscillation

**Figure 3:**
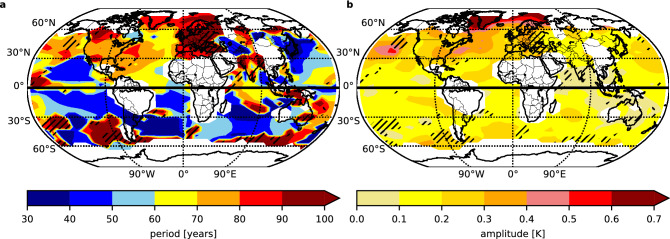
Global distributions of periods and amplitudes for the strongest oscillation in the period range > 30 years. (**a**), (**b**) The data have been averaged in 8° × 4° longitude-latitude boxes. Only results for average values with full temporal coverage for all single data points have been considered. White areas show regions, where not enough or no data are available. The hatched areas mark region where the data were fitted sequentially.

To get insight into the hemispheric differences of the strongest oscillation, we derived a global distribution of the periods and amplitudes. We focused on the period range larger than 30 years to obtain results for the strongest oscillation only and omitted the quasi-bidecadal oscillation. The long-term trend is described by a second-degree polynomial. Figure 3 shows the global distributions of the period and amplitude of this oscillation. The period shows clear hemispheric differences in the same way as the results for the hemispheric mean time series. In the NH the period is predominately larger than 60 years (yellow to red colours) with only some regions showing periods in the range 30–60 years (blueish colours; mostly over sea). In SH, the situation is reversed and the regions where a period in the lower range was identified are predominant. The global distribution of the amplitude also reflects the behaviour observed for the hemispheric mean time series. Larger amplitudes in the NH than in the SH and a large increase of the amplitude towards the Arctic can be seen. For example, the amplitude in the region of Greenland reaches values larger than 0.7 K, which is more than twice as large as in Europe and North America. In the SH the amplitudes only reach values of 0.1 K, for example in parts of Australia. This additionally underlines the importance of the NH for the evolution of the global mean surface temperatures.

## Comparisons with other data sets

To ensure that these results are not an accidental phenomenon or influenced by the choice of the data set, we additionally analysed other data sets. For this purpose we made use of the HadCRUT5 Analysis from the Met Office Hadley Centre and the Climatic Research Unit at the University of East Anglia^30–32^, the NOAA Merged Land Ocean Global Surface Temperature Analysis (NOAAGlobalTemp v5)^33,34^, and the Japan Meteorological Agency’s (JMA) Global Surface Temperature Anomalies.

In the case of the global annual mean temperature anomalies, all three data sets exhibit the strongest oscillation in the period range between 66 and 74 years with amplitudes from about 0.089–0.123 K. These results are in good agreement with the results obtained before. The data sets also show clear hemispheric differences for the periods and for the amplitudes. In the NH the periods range from about 74 to 79 years and are slightly smaller than those for GISTEMP v4, with HADCRUT5 showing the largest difference. The periods in the SH range from about 48 to 65 years (JMA’s Global Surface Temperature Anomalies show the largest difference). The larger spread of the results in the SH likely stem from the data sets used for the sea surface temperatures (SST), because the ocean covers a large part of the SH. Another indication is the good agreement between the GISTEMP v4 dataset and NOAAGlobalTemp v5, with almost identical results for the periods. Both datasets use the Extended Reconstructed Sea Surface Temperature Version 5 (ERSST5)^34,35^. Moreover, the hemispheric difference for the amplitudes is also clearly observed for the data sets. The amplitudes in the NH range from about 0.157 to 0.178 K and are more than twice as large as the amplitudes in the SH (about 0.065–0.072 K).

## Impact of strongest oscillation on temperature increase

The long-periodic oscillations largely modulate the observed increase or decrease of the surface temperatures. This phenomenon of a fluctuating temperature increase as a result of multi-decadal temperature fluctuations has already been shown by other authors^5,15,16^. However, an important question is how large the anthropogenic and the natural contribution to the observed overall temperature increase are. Tung and Zhou argue that the observed strongest long-period oscillation is natural because it dates back to the pre-industrial era and therefore cannot be forced by anthropogenic aerosol in the industrial era^16^, as suggested by other studies^24,25^. As shown above, the strongest long-periodic oscillation remains when a regression using the radiative forcing that includes all human influenced contributions is applied. Therefore, we agree that the oscillation is very likely of natural origin. Consequently, the two different parts of the curve (long-term trend and sine curve) can be seen as an anthropogenic and natural contribution, respectively. Because of the good agreement between the results for regression and second-degree polynomial in the later part of the time series (see Fig. 1a), both descriptions should give an approximate separation between the anthropogenic and the natural shares.

Figure 2a illustrates the two different contributions to the observed total increase for one specific example. The orange horizontal lines show the mean values of the second-degree polynomial in the time intervals 1971–1980 and 2009–2018, respectively. The mean values of the complete fit in the same time intervals are shown as green horizontal lines. Obviously, the temperature increase from the first to the second interval is larger for the complete fit than for the polynomial only, since the oscillations positively add to the long-term trend. The differences (vertical green and orange lines in Fig. 2a) give the contribution of the sinusoids, i.e., the approximate natural variability, to the total temperature increase. In this case, the contribution is about 20%. In absolute terms, the total increase is about 0.70 K, with the contribution from the natural variability being about 0.14 K and the anthropogenic contribution being about 0.56 K. The second strongest oscillation with a period of about 20.6 years plays only a minor role here and the contribution only changes to 23% when excluded. Using the regression results, the contribution is about 19%, which shows the good agreement of these two descriptions. Using other time intervals, especially in the period of greatest change in aerosol forcing, may increase the differences between the two descriptions. Here, the aerosol contribution to radiative forcing leads to a reduction in the overall observed increase (cf. Figure 1a), and the two descriptions are therefore not perfectly comparable. In completely different time intervals than those chosen in Fig. 2, the influence of the strongest oscillations on the observed temperature increase can also be negative, i.e., the oscillations reduce the observed temperature increase. This can be, for example, observed in the time intervals from 1941–1950 to 1971–1980. Because of the large hemispheric differences in the period and especially the amplitude of the strongest oscillation, the contribution to the total increase from 1971–1980 to 2009–2018 is also significantly different. In the NH, the contribution enlarges to about 29%. Whereas, in the SH, due to the much smaller period, the contribution is negative, with a value of about − 16%, i.e., it reduces the larger human-induced temperature increase that probably exists.

**Figure 4:**
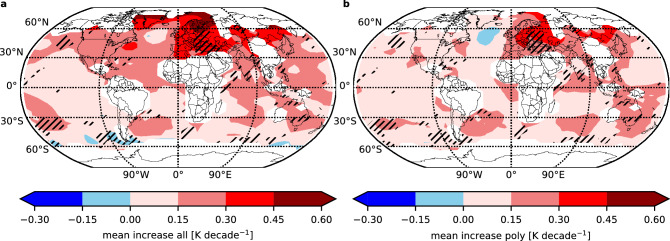
Difference between observed total temperature increase and approximative anthropogenic part. (**a**), (**b**) Global distributions of the mean temperature increase from the time interval 1971–1980 to 2009–2018 for the full increase (**a**) and the polynomial only (**b**), which approximatively shows the anthropogenic part. Only results for average values with full temporal coverage for all single data points have been considered. White areas show regions, where not enough or no data are available. The hatched areas mark region where the data were fitted sequentially.

Figure 4 shows the temperature evolution in more detail. The figure shows the mean temperature increase from the midpoint of the 1971–1980 time interval to the midpoint of the 2009–2018 time interval for the entire fit (Fig. 4a) and for the polynomial only (Fig. 4b), expressed as an increase in K decade^−1^. Clearly visible is a strong reduction of the increase in the NH when we subtract the oscillation. Sometimes it even turns out that the long-term trend is slightly negative and only the strongest oscillation turned it positive (e.g., over the North Atlantic). The effect is greatest in Greenland, where the amplitude is largest. The next region is Europe with slightly larger effects than in North America.

In the SH the effects are much smaller and partly positive and partly negative. Thus, this analysis additionally demonstrates the importance of the NH to the evolution of surface temperatures and the variation in observed temperature increases over different time intervals.

## Discussion

Our results for the periods of the strongest oscillations determined for the global mean surface temperatures agree well with recent publications, where also multi-decadal variations with a period of about 65–70 years and a quasi-bidecadal oscillation have been reported^4,5,15^. The amplitudes of 0.1 and 0.04 K reported for the approximately 70-year and quasi-bidecadal oscillations, respectively^5^, also show reasonable agreement. In addition to this analysis of global mean temperatures, we also analysed hemispheric and regional differences in both period and amplitude. In former studies the regional differences in periods were not directly considered and only a regression analysis using the global results and the globally resolved temperature field was performed^5,15^. Therefore, a locally smaller response in the regression analysis can be either due to a smaller amplitude of the oscillation or a locally not matching period. However, the significant increase in the amplitude of the strongest oscillation (about 70 years) toward the North Pole is clearly observed in former studies^5,15^. Model simulations and observations showing increasing low-frequency variability toward the North Pole^36^ further confirm this result.

The larger overall temperature increase in the high northern latitudes (see Fig. 4a) is also in good agreement with studies of Arctic amplification (AA), the larger near-surface temperature change in Arctic regions compared to lower latitudes and global averages^37^. However, the influence of the strongest long-period oscillation on the temperature increase plays an important role here, as the analysed time interval is a crucial parameter. In a recent review study, the 1979–2018 time interval is used in many comparisons and analyses^37^. This time interval goes from about minimum to maximum of the long-period oscillation (e.g., Fig. 2). Because of the oscillation, the temperature change is largest, and would be significantly different in other time intervals. Other authors also showed that linear trends in, for example, Greenland and the northern part of North America are largest in the time interval 1981–2017, but much smaller in other time intervals^38^. Furthermore, this may explain some of the observed differences between observations and model simulations, where simulations show less warming in the Arctic lower troposphere when the 1979–2018 period is analysed^37^. If the models underestimate or incorrectly simulate the long-period oscillation, this would result in an underestimation of the total temperature increase. However, one must be very careful when analysing and interpreting temperature increases in time intervals shorter than the period of possible long-period oscillations. This is, for example, very obvious in the North Atlantic region, where it turned out that the long-term trend is even slightly negative and only the long-period oscillation turned the total temperature increase positive. The region seems to be nearly completely dominated by the long-period oscillation and the anthropogenic influence on the temperatures seems to be very small. This finding is in good agreement with another study where a regression of the global sea surface temperature field on the secular trend (long-term trend) also showed slightly negative values, i.e., the long-term trend in this region is slightly negative^15^.

Several studies state that the observed multi-decadal variability of global surface temperatures is caused or, at least, largely influenced by the AMO/AMV or IPO/PDO^5,16,20–23^, but there are largely different opinions on that. For example, Meehl et al. and Dai et al. state that the IPO is largely responsible for the observed slow down and acceleration of the temperature increase, i.e., for the multi-decadal variability of the surface temperatures^20,21,23^. Other studies suggest a combination of PDO and AMO as cause for the temperature fluctuations^22^. On contrast to that, Chen et al. argue that the influence of the Pacific (IPO/PDO) on the global mean temperature variation in the multi-decadal time scale is very small compared to the larger influence of the AMO^39^. Other authors also state that the multi-decadal variability is mainly caused by the AMO^5,16^. Since the period of the strongest oscillation is very different at different locations on the globe, we believe that such an explanation alone is not sufficient to fully explain the observations. With regression analysis other authors observed a very strong response over the North Atlantic and North America, but not over Europe^15^. This could be caused by a mismatched period of the oscillation over Europe compared to the global period used for the regression analysis. Our study clearly shows this mismatch between Europe on one side and the North Atlantic, North America and the global mean on the other side (see Fig. 4). In an older study such differences between the North Atlantic (about 76 years) and North America and Eurasia (about 88 years and 84 years, respectively) have also been observed, but with a larger difference to North America^12^. Thus, in our view, an explanation for the magnification of the period in parts of the NH compared to the global mean or the North Atlantic region is missing. Nonetheless, the good agreement between the period of the AMO index at about 70 years^5^ and the observed period for the strongest oscillation of the surface temperatures in the North Atlantic may be indicative for a role of the AMO in this process. A complete clarification of this issue requires additional analyses that are beyond the scope of this paper. Apart from these differences, we also agree that the observed strongest oscillations are natural in origin, as do other studies^4,5,15,16^. Since the oscillation period and amplitude remain constant when a regression using radiative forcing is performed, the cause of this oscillation is most likely natural. Thus, most of the multi-decadal variability is due to natural variability rather than human-induced aerosols in the atmosphere, as suggested by other studies^24,25^. In a previous study we observed similar oscillations in simulations with fixed boundary conditions, i.e., no increase in greenhouse gas concentrations, no long-term changes of the sea surface temperatures and solar irradiance^11^. This shows that there seems to be no direct influence of these parameters on the oscillations, which gives a first hint that there must be other excitation mechanisms. Thus, the authors believe that the oscillations might be self-excited in the atmosphere. In the observations a combination of both, external forcing (e.g., by the ocean or sun) and the capability of the atmosphere itself to generate internal oscillations is imaginable.

Nonetheless, aerosol forcing may contribute also to the slowdown in temperature increase after 1940, as indicated by the slowdown in the increase in radiative forcing during this period (Ref.^29^ and Fig. 2a). However, an oscillation is necessary to explain the overall behaviour of the temperature curve, as the slowdown in radiative forcing alone is too small.

A cause for the hemispheric differences for the oscillations cannot be easily named, but as the oscillations in the period 30–60 years mainly occur over sea surfaces (see Fig. 3a), this may give a hint. Because the land to ocean proportions are largely different in the NH and SH and much more sea surface exist in the SH, this may influence the observed periods and, at least partly, explain the observed differences.

## Methods

### Data

The temperature data set mainly used in this study is the GISS (Goddard Institute for Space Science) surface temperature analysis version 4 (GISTEMP v4). In this data set the observations of meteorological stations over land (NOAA GHCN v4) are combined with sea surface temperatures at ocean areas (ERSST v5)^26–28^. The temperature data are anomalies with respect to the base period 1951–1980. They are available as global and hemispheric mean values as well as globally resolved temperature anomalies on a 2° × 2° grid. The data cover the period from 1880 to the present. Here we only use full years and therefore the data series ends in 2020. In the case of the globally resolved dataset, there are regions where the time series starts later due to lack of measurements, e.g., Antarctica and Africa. We excluded this regions from our analysis. The temperature anomalies are provided with a monthly temporal resolution. In our case we only use annual averages because we are only interested in long-term behaviour and want to exclude the typical seasonal cycle from the analysis.

For comparisons other global data sets were analysed, namely HadCRUT5 Analysis from the Met Office Hadley Centre and the Climatic Research Unit at the University of East Anglia^30–32^, the NOAA Merged Land Ocean Global Surface Temperature Analysis (NOAAGlobalTemp v5)^33,34^, and the Japan Meteorological Agency’s (JMA) Global Surface Temperature Anomalies.

### Time series of radiative forcing

We use the time series of CO_2_ equivalents (CO_2_e) made available by the European Environment Agengy (EEA)^40^. The time series includes all relevant greenhouse gases (Kyoto and Montreal Protocol) as well as further gases (O_3_, water vapour), aerosol and other forcing contributions. For the greenhouse gases the CO_2_e is calculated using the approximate equations according to IPCC^29,41^. The further forcing contributions such as aerosol, O_3_ and water vapour are mainly from IPCC^29^ and related studies that analyse the evolution after the report. More details about the time series can be obtained from EEA^40^. We used the same approximate equations according to IPCC^29,41^ to transform the CO_2_e back to radiative forcing in Wm^−2^. The time series of radiative forcing covers the period from 1860 to 2018.

## Methodology

### Description of the long-term trend

**Figure 5:**
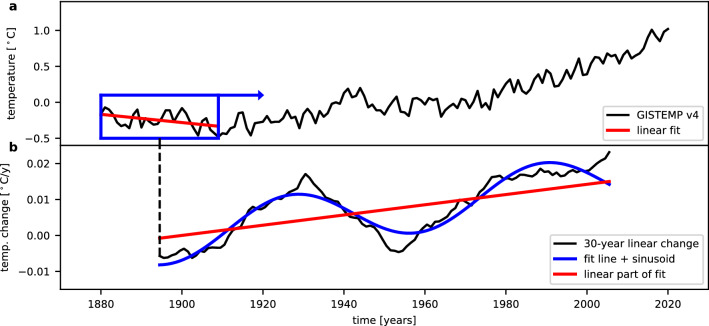
Long-term evolution of global annual mean surface temperatures. (**a**) The global annual mean surface temperature anomalies are shown as black curve. The blue box shows the first 30 year window used for the estimation of the linear temperature changes. Inside the box the result of the linear regression is displayed as red line. (**b**) The linear temperature changes (slopes of linear regression) in consecutive 30 year windows are shown as black curve at the mean times of the corresponding time window. The blue curve shows the result of a least squares fit of a line together with a sinusoid to the time series. The red line shows only the linear part of the same fit.

The time series of the surface temperature anomalies (black curve in Fig. 5a) shows a long-term trend, i.e., an overall increase of the temperatures, and superimposed oscillations. In order to determine the parameters of the oscillations the long-term trend needs to be sufficiently described. There are several ways to describe this trend, such as a line, a polynomial of second or higher degree, an exponential increase, or other curves. A way to gain more information on the long-term trend can be obtained from the derivative of the time series. Figure 5b shows the linear temperature changes (slopes of linear regression) in consecutive 30 year windows, i.e., a smoothed derivative of the temperature time series. This derivative does not only show the large variations in the linear temperature changes as already presented before^15^, it additionally yields information on the time series itself. First, the oscillation is clearly visible as the differentiation only shifts the phase of an oscillation but keeps the period. Second, the temperature changes still show an overall increase with time (see red line in Fig. 5b). This means that a line, which would be the most simple description of the long-term trend, is not advisable. If the original temperature time series could be described by a line plus a sinusoid, only the phase shifted sinusoid and a constant offset would remain after differentiation. This is obviously not the case. However, the temperature changes can sufficiently be described by a linear term plus a sinusoid (see blue curve in Fig. 5b). This means, that the original time series can be described with a second degree polynomial plus a sinusoid in the simplest way. A second possibility to describe the long-term trend is the use of an exponential function. Since the surface temperatures T_S_ certainly depend on the radiative forcing (RF), which can be approximated by

$$\Delta {\text{T}}_{{\text{s}}} \approx\uplambda \cdot {\text{RF}},$$

where λ is the climate sensitivity^42^, the long-term trend can also be described by this relationship, i.e., by the regression with the radiative forcing. In the analysis all three possibilities will be used and compared.

### Analysis of periodicities

We analyse the periodicities with a combination of least squares fitting and periodogram analysis. In order to obtain a good first guess for the period and amplitude of the main oscillation, the temperature time series is first detrended by subtracting a fit describing the long-term trend. For the residual temperatures, the Lomb-Scargle periodogram (LSP)^43,44^ is determined in the period range 20–141 years to find the strongest long-period oscillations. As the LSP is equivalent to least squares fitting of sinusoids^45^, the maximum peak shows the period that leads to a sinusoid with maximum amplitude in a fit, i.e., the strongest oscillation. This maximum peak then defines the initial values and a sinusoid plus the long-term trend are fitted together. Further possible oscillations can be determined in the same way. The complete fit including long-term trend and main oscillation is subtracted from the original time series. The residual temperatures are again analysed using the LSP to find the next maximum peak, i.e., the next strongest oscillation. This sequential procedure is done to ensure that the second peak is not an artifact or spectral leackage influenced by the first maximum peak. The next oscillation then is valid under the assumption that the first oscillation is also real^45^. In a last step then all components are fitted together (two sinusoids and the long-term trend). We are here interested in the one or two strongest oscillations. In the case of the globally resolved analysis for some situations, the convergence of the fit is not good and the difference of the determined period to the initial guess becomes very large. In such cases partly a replacement of the second-degree polynomial by a line is sufficient to obtain a stable fit. In the remaining cases (about 11%) the fitting is done sequentially, i.e., first the data have been detrended and then the sinusoid is fitted to the residual temperatures. These data points are marked by the hatched areas in the figures.

The significance of peaks in a LSP can be evaluated using the false alarm probability (FAP). The FAP gives the probability that a peak of a certain height can occur just by chance, e.g., by noise^46–48^.

## Data Availability

The temperature data have been provided by the Goddard Institute for Space Science and can be downloaded from the site https://data.giss.nasa.gov/gistemp/. The data set of equivalent CO_2_ has been provided by the European Environment Agency and can be obtained from the site https://www.eea.europa.eu/data-and-maps/indicators/atmospheric-greenhouse-gas-concentrations-7/assessment/.
